# Healthcare analytics perspectives on artificial intelligence–based retinopathy of prematurity screening: a bibliometric mapping study

**DOI:** 10.3389/frma.2026.1772126

**Published:** 2026-04-30

**Authors:** Amina Turki, Nazar Salih Abdulhussein, Rasha W. Mohammed Taher, Mohamed Ksantini

**Affiliations:** 1 Control and Energies Management Laboratory (CEM-Lab), National Engineering School of Sfax, University of Sfax, Sfax, Tunisia; 2 Computer Science Department, Al-Imam Al-Adham University College, Baghdad, Iraq; 3 Human Resources Department, Ministry of Higher Education and Scientific Research, Administrative and Financial Affairs Directorate, Baghdad, Iraq; 4 Advanced Technologies for Image and Signal Processing (ATISP) Lab, ENET'Com & IPEIS, University of Sfax, Sfax, Tunisia

**Keywords:** artificial intelligence, bibliometric analysis, deep learning, medical screening, Middle East, retinopathy of prematurity

## Abstract

The integration of artificial intelligence (AI) into retinopathy of prematurity (ROP) screening represents an important advancement in neonatal ophthalmology, particularly in settings facing specialist shortages and screening infrastructure constraints. Despite the rapid expansion of research in this domain, a structured evaluation of its intellectual landscape, geographic distribution, and collaboration patterns remains limited. This study presents a bibliometric mapping analysis of global research on AI-based ROP screening. A total of 55 publications indexed in the Scopus database were identified through a reproducible search strategy and analyzed using Bibliometrix and VOSviewer. The retrieved publications span the period 2017–2025. The results demonstrate a marked upward trajectory in publications, with a peak of 16 publications in 2025 and an estimated annual growth rate of 41.42%. The analysis examines publication trends, geographic productivity, institutional collaboration networks, citation patterns, and thematic evolution. The findings indicate strong contributions from Asia and increasing participation from the Middle East, particularly Türkiye, Saudi Arabia, and Egypt. International co-authorship networks reveal structured South–North collaborations linking Middle Eastern countries with major research hubs in the United States and the United Kingdom. Citation analysis shows an average of 18.27 citations per publication. Thematic mapping highlights the dominance of deep learning and retinal image analysis, alongside emerging discussions on healthcare accessibility and deployment in resource-limited settings. These results provide a structured quantitative overview of the development of AI-based ROP screening research and may support future research prioritization and strategic planning in AI-assisted neonatal eye care.

## 1 Introduction

Retinopathy of prematurity (ROP) is a vasoproliferative disorder affecting the developing retina of premature infants and remains a leading cause of preventable childhood blindness worldwide (Kourosh et al., 2022). The global burden of ROP is evolving, with increasing rates observed in middle-income countries due to improved survival of preterm neonates. Indeed, it continues to be a significant concern in neonatal intensive care units globally (Sumer et al., 2025).

Globally, an estimated 13–15 million preterm infants are born each year, with retinopathy of prematurity remaining one of the leading causes of preventable childhood blindness. The burden is particularly pronounced in middle-income countries, where improvements in neonatal survival have not been matched by proportional expansion in screening infrastructure and specialist availability. This imbalance underscores the need for scalable and technology-assisted screening solutions.

The current standard for diagnosis relies on serial dilated binocular indirect ophthalmoscopy examinations by skilled ophthalmologists, a process that is resource-intensive, subjective, and logistically challenging, especially in regions with a shortage of specialists (Song et al., 2026).

The integration of Artificial Intelligence (AI) into healthcare has ushered in a new era of medical diagnostics, offering capabilities in image analysis, pattern recognition, and predictive modeling (Esteva, 2019; Prasanna et al., 2025; Liu et al., 2021). It becomes increasingly fundamental in ophthalmic diagnostics, revolutionizing disease detection through Deep Learning (DL) and image analysis techniques. Recent advances have enabled AI systems, particularly DL (Dash et al., 2024; Acevedo et al., 2025), to reach ophthalmologist-level performance in detecting and grading retinal diseases, including diabetic retinopathy and age-related macular degeneration (Li et al., 2024; Vidivelli et al., 2025).

These innovations provide the groundwork for applying similar methodologies to ROP which represents a major milestone in neonatal ophthalmology. Deep learning models have been successfully trained to identify plus disease detection, classify disease severity, and even predict its progression from retinal images with acceptable accuracy (Brown et al., 2018; Campbell, 2023). These technological advancements promise to enhance screening efficiency, improve accessibility, and standardize diagnosis, by mitigating the risk of vision loss (Kiran Yenice et al., 2024; Peijie et al., 2025; Madhu et al., 2024).

The rapid advancement of this research area indicates that scientific literature has become vast and multifaceted. A systematic structured consolidation of this topic is therefore essential to map the intellectual landscape, identify key research trends, and highlight collaborative networks. While traditional systematic reviews summarize existing research, bibliometric analysis provides a complementary, data-driven perspective by quantifying publication performance, mapping scientific networks, and screening the conceptual and social structure of a specific research domain. This big-data–oriented approach enables a comprehensive level of the evolution, influence, and dynamics of the field (Marlés-Sáenz et al., 2025).

**Figure 1:**
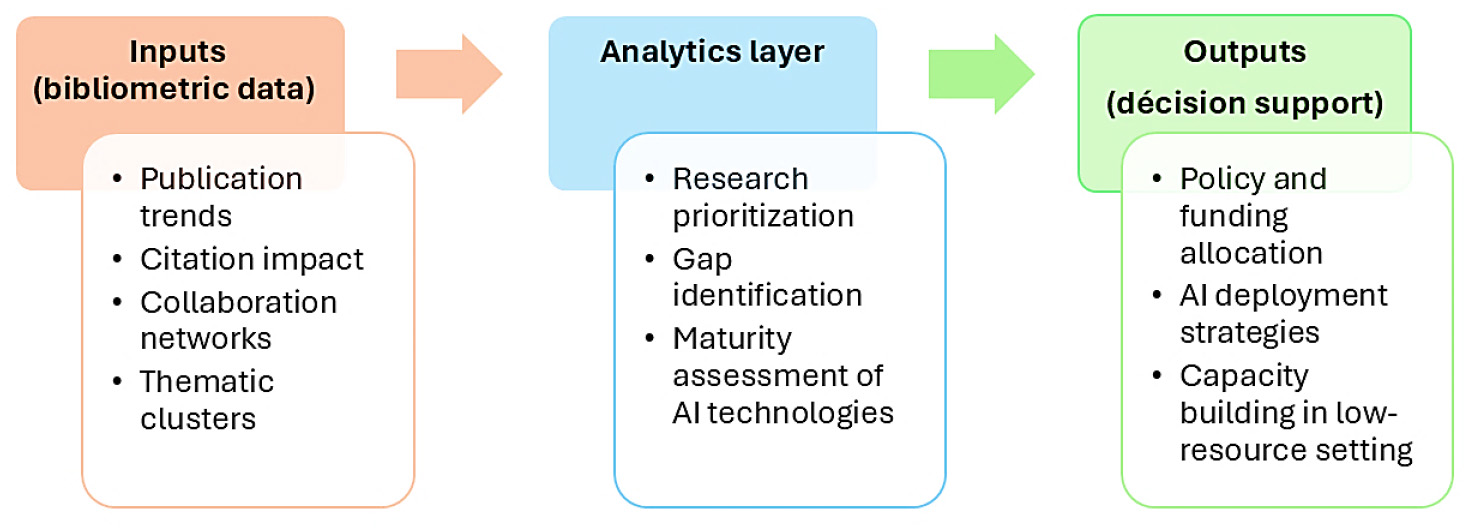
Conceptual framework illustrating the relationship between bibliometric findings and healthcare planning.

From a healthcare analytics perspective, bibliometric analysis is considered as a strategic tool rather than a descriptive method. It systematically quantifies research productivity, and collaborations to provide bibliometric indicators inputs and perceptions that support evidence-based decision-making in healthcare systems (Figure 1).

Recent bibliometric publications indicates that research on AI in medicine and health has surged in the Eastern Mediterranean Region, with Saudi Arabia among the leading contributors, highlighting a growing regional engagement with digital-health innovation (Al-Dekah, 2025). Another bibliometric data illustrates that ophthalmology research output from Arab-affiliated institutions has more than doubled over the past decade (2012–2022), with Egypt and Saudi Arabia leading the region in publication rate (Yu et al., 2024). Given the global strategy toward AI-integration in ophthalmology, this needs a growing capacity and scientific interest in AI-ophthalmology within these countries.

AI-based ROP screening has particular attention not only in high-resource settings but also in low- and middle-income regions, where its evolution can help overcome specialist shortages (Ortiz et al., 2025). Despite the growing volume of AI-based ROP research, no inclusive bibliometric assessment in this context has yet mapped its global evolution and intellectual structure. This growing regional activity underscores the need for bibliometric mapping to recognize collaborative patterns and research impact in this domain.

Several bibliometric studies have dealt with AI in ophthalmology (Ting et al., 2019). These works mainly focused on cataract and diabetic retinopathy research (Deng and Qin, 2025; Zhao et al., 2025; Yu et al., 2024). A bibliometric analysis (Gao et al., 2025) that mapped AI-based research on ROP over the 2010–2023 period, highlights thematic trends in the field. However, the study did not explore geographical contributions or international collaboration networks. Therefore, a decade-long bibliometric analysis is needed to map publication trends, collaboration networks, and conceptual themes, highlighting Middle Eastern contributions.

While previous bibliometric studies have examined artificial intelligence applications in ophthalmology more broadly, and some have explored thematic developments in AI-based ROP research, limited attention has been directed toward detailed mapping of international collaboration networks and regional participation patterns specific to AI-based ROP screening. The present study addresses this gap by providing a focused geographic and collaboration-centered bibliometric mapping.

This study provides a comprehensive bibliometric analysis of global research on AI-based ROP screening from 2015 to 2025, with specific contributions from the Middle East. This bibliometric analysis highlights key contributors, collaborative networks, and emerging conceptual clusters. Through this work, we seek to illuminate how AI is reforming neonatal ophthalmology and to situate the Middle East's growing role within this transformative field.

This paper methodology consists on:

(1) quantify the growth trajectory and publication trends,
(2) identify the most influential countries, institutions, authors, and journals,
(3) map the conceptual structure through keyword co-occurrence analysis,
(4) visualize the international collaboration networks.

The findings will provide valuable reference for researchers, clinicians, and policymakers to understand the current state and future directions of this transformative field.

Thus, this paper is organized as follows: section 2 describes the data source and the bibliometric methodology adopted in this study. Section 3 presents the main findings in terms of publication trends, geographical distribution and collaboration networks of the research topic. Section 4 discusses these findings and highlights key insights to address the study's limitations. Finally, the conclusion summarizes the main contributions and draws directions for future research.

## 2 Materials and methods

### 2.1 Study design

This study is a bibliometric mapping analysis rather than a systematic review of clinical outcomes. The objective was to quantitatively map publication trends, collaboration networks, and conceptual structures within the field of AI-based ROP screening.

Accordingly, the methodology follows bibliometric analysis standards and does not adopt a PICOS-based evidence synthesis framework or full-text critical appraisal of clinical studies.

### 2.2 Data source

This bibliometric analysis was conducted exclusively using the Scopus database. Scopus was selected due to its extensive multidisciplinary coverage and comprehensive citation metadata, which are particularly suitable for bibliometric mapping and network analysis (Jeroen et al., 2020).

No additional databases were searched, as the objective of this study was to perform a structured bibliometric analysis within a single, standardized citation index to ensure data consistency (Powell and Peterson, 2017; Alryalat et al., 2019).

### 2.3 Search strategy

The search was conducted on September 17, 2025 using a structured combination of keywords related to the disease (“*retinopathy of prematurity” OR “ROP”*), the applied technology (“*artificial intelligence” OR “machine learning” OR “deep learning”*), and the application context (“*screening” OR “diagnosis”*). Boolean operators and truncation symbols were employed to ensure comprehensive coverage while maintaining precision.

The inclusion criteria were:

- **Publication years:** 2015 – 2025 (inclusive)
- **Document type:** Articles and reviews
- **Language:** English

All search results were exported with complete bibliographic metadata - including titles, abstracts, author information, citations, and keywords - in CSV format for subsequent analysis.

### 2.4 Search strategy and reproducibility

To ensure full transparency and reproducibility of the dataset, the exact Scopus search query executed in this study is provided below. The search was conducted in the Scopus database on September 17, 2025.

The search string was applied to the TITLE-ABS-KEY fields as follows:

(TITLE-ABS-KEY(“retinopathy of prematurity” OR “ROP”) AND TITLE-ABS-KEY(“artificial intelligence” OR “machine learning” OR “deep learning”) AND TITLE-ABS-KEY(“screening” OR “diagnosis”)) AND (PUBYEAR > 2014 AND PUBYEAR < 2026) AND (LIMIT-TO(DOCTYPE, “ar”) OR LIMIT-TO(DOCTYPE, “re”)) AND (LIMIT-TO(LANGUAGE, “English”))

For enhanced transparency and reproducibility, the search strategy is summarized in Table 1. The table presents the full query string, database source, search fields, applied filters, and the total number of records retrieved at the initial search stage prior to screening.

**Table 1:** Search strategy summary.

Query string	Database	Search fields	Year range	Document type	Language	Initial records retrieved
TITLE-ABS-KEY(“retinopathy of prematurity” OR “ROP”) AND TITLE-ABS-KEY(“artificial intelligence” OR “machine learning” OR “deep learning”) AND TITLE-ABS-KEY(“screening” OR “diagnosis”)	Scopus	Title, abstract, keywords	2015–2025	Articles, Reviews	English	75

As shown in Table 1, the structured query applied in the Scopus database yielded 75 records prior to duplicate removal and relevance screening.

The search was restricted to articles and review papers published between 2015 and 2025 (inclusive) and limited to English-language publications. Although the search window covered 2015–2025, the retrieved publications spanned the period 2017–2025.

All retrieved records were exported in CSV format with full bibliographic metadata, including authors, affiliations, abstracts, keywords, and citation counts.

Screening was conducted at the title and abstract level to confirm relevance to artificial intelligence–based screening or diagnosis of retinopathy of prematurity. No full-text eligibility assessment was performed, consistent with bibliometric mapping methodology.

### 2.5 Data screening and preparation

A total of 75 records were identified through the Scopus database search. After removing 5 duplicate records, 70 unique publications remained.

During the title and abstract screening stage, 15 records were excluded due to lack of relevance to artificial intelligence–based screening or diagnosis of retinopathy of prematurity.

Consequently, 55 publications met the inclusion criteria and were included in the final bibliometric analysis.

**Figure 2:**
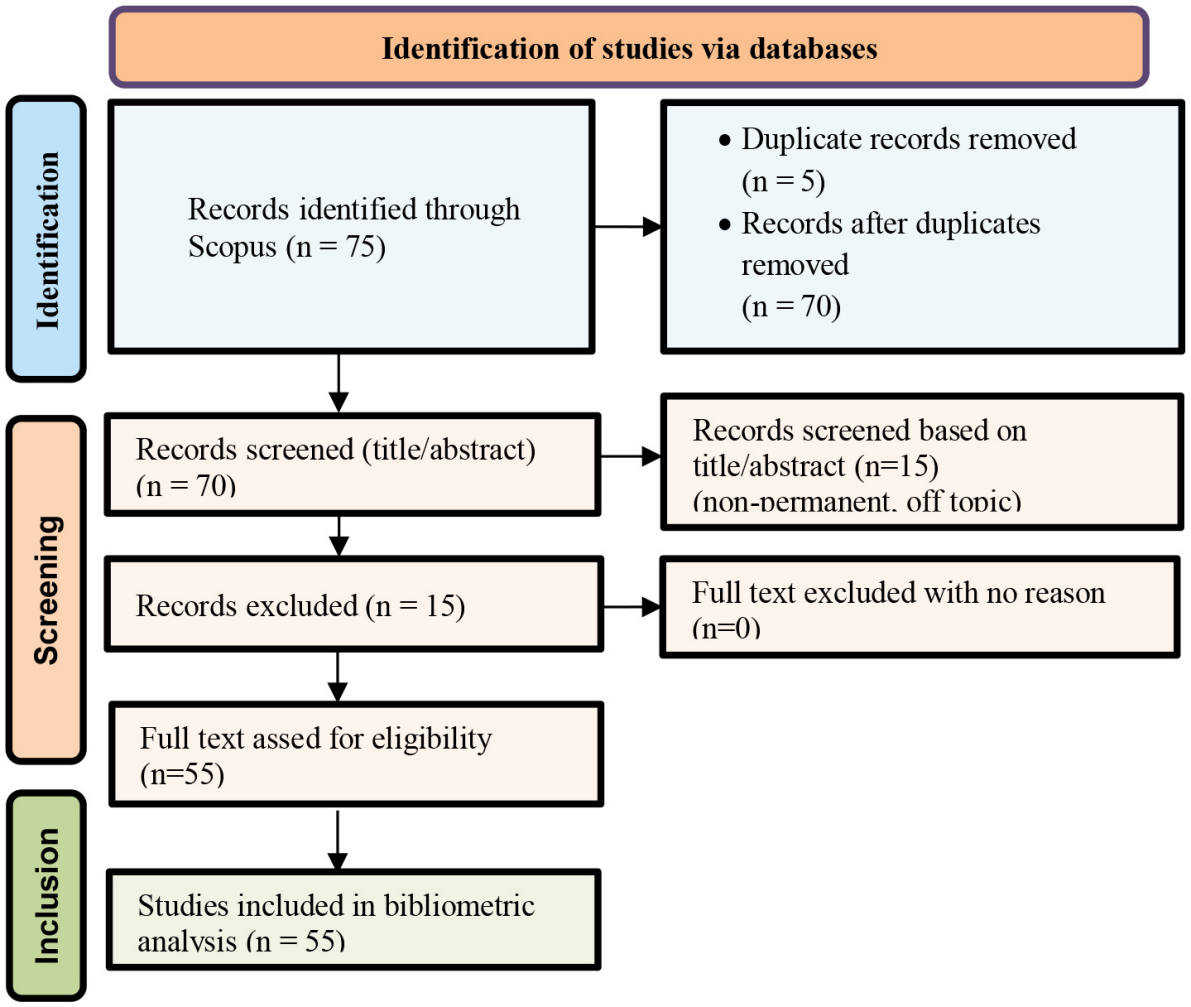
Flow diagram of database search and screening process.

The screening workflow is summarized in Figure 2.

### 2.6 Bibliometric analysis

#### 2.6.1 Analytical tools and workflow

The cleaned and refined dataset, comprising 55 publications, was exploited to a comprehensive bibliometric analysis using a dual-tool approach to ensure robustness and depth analysis. It was analyzed using an integrated bibliometric workflow combining *Bibliometrix* (v. 4.3.0) (Aria and Cuccurullo, 2017), and *VOSviewer* (v. 1.6.20) (Van Eck and Waltman, 2010).

- **Bibliometrix**, an R-tool package and its web interface Biblioshiny, were used for a primary quantitative and performance analysis. Key metrics such as annual publication growth, most productive sources, authors, and institutions were extracted.
- **VOSviewer** was employed for advanced network construction, visualization and science mapping to generate network structures and produce detailed visualizations for the science mapping component of the study, including co-authorship, co-citation, and keyword co-occurrence networks.

This workflow ensured a systematic and comprehensive examination of publication patterns and research relationships within the field.

#### 2.6.2 Dimensions of analysis

The analysis was conducted across three primary dimensions:

##### 2.6.2.1 Performance analysis

This aspect of the analysis assessed both the scientific productivity and the scholarly impact of research in the field. Numerous performance indicators were examined. Annual publication trends were analyzed to trace the field's growth relationship and temporal development. Many productive sources were identified to highlight the journals that contribute most prominently to research on AI-based ROP screening. Leading authors and affiliations were drawn to recognize major contributors and influential organizations, with particular attention to research emerging from the Middle East. The geographical distribution of output was done by identifying the most productive countries involved in this research area. Finally, citation-based indicators, including the average number of citations per document and per year, were used to evaluate the visibility and the academic impact of the published literature.

##### 2.6.2.2 Conceptual structure

This part of the analysis explored the intellectual landscape and thematic growth of the research area. The main approach employed for this part was keyword co-occurrence analysis. Author keywords were quantified, and a network was created in which the links between terms revealed how frequently they appeared together within the same documents. By using VOSviewer for visualization, this network could; (i) identify the central research themes, (ii) understand how they relate to one another, and (iii) outline the theoretical structure of the field.

##### 2.6.2.3 Network analysis

This step focused on collaboration relationships among researchers and institutions. Co-authorship analysis was achieved by making networks between both the country and institutional levels, allowing visualization of international and national partnerships. This approach highlighted key collaborative hubs and relationships of knowledge exchange, with prominence on contributions from the Middle East. All visual outputs, including conceptual networks (keyword co-occurrence maps), social networks (collaboration maps), and production trends (annual publication histograms), were generated right from the analysis tools to safeguard both accuracy and reproducibility.

#### 2.6.3 Reproducibility and data availability

All search parameters, analysis texts, and outputs can be replicated using the Scopus database with the provided request to ensure transparency. No patient data or human subjects were involved in this study, and all the analyzed data are publicly available through Scopus. All subsequent analyses, including co-authorship networks and citation patterns, are based on this preserved set of studies.

## 3 Results and discussion

### 3.1 Annual publications per year (2015-2025)

The annual publication trend provides a key indication of how research interest in applying AI to ROP screening has grown over time.

**Figure 3:**
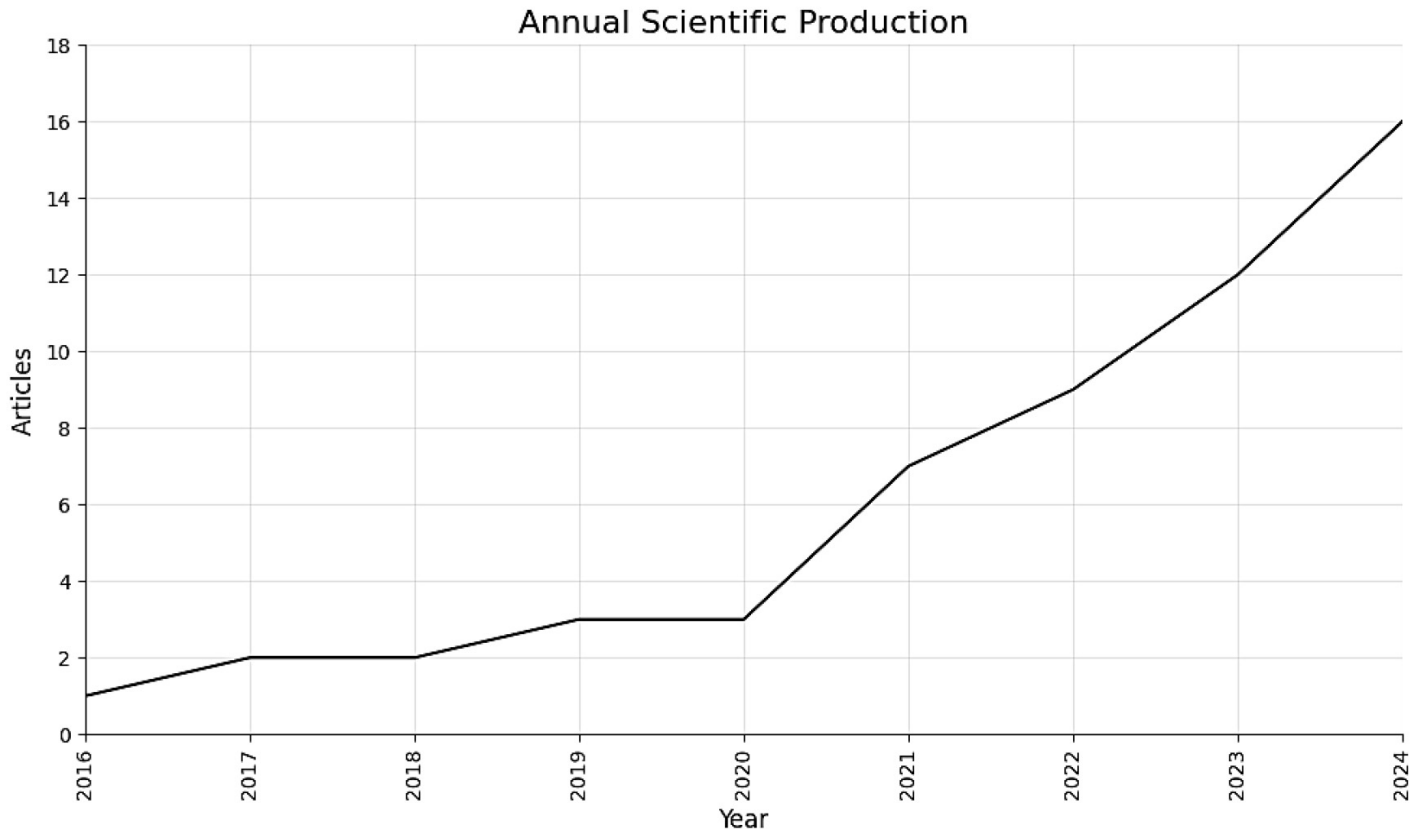
Annual Scientific Production related to AI in ROP Screening (2015–2025).

As shown in Figure 3, research activity in this domain has followed a clear upward trend since the first indexed publications in 2017. Output remained modest until 2019, after which a steady and sustained increase was noted, reaching a peak of 16 publications in 2025. This expansion corresponds to an estimated annual growth rate of 41.42%, reflecting a consistent increase in publication output over the study period.

The sharp rise in publications from 2020 was justified by overall progress in deep learning methods and the wider adoption of digital retinal imaging, two factors that have facilitated AI-based approaches to ROP screening.

### 3.2 Annual publications by country (2015-2025)

**Figure 4:**
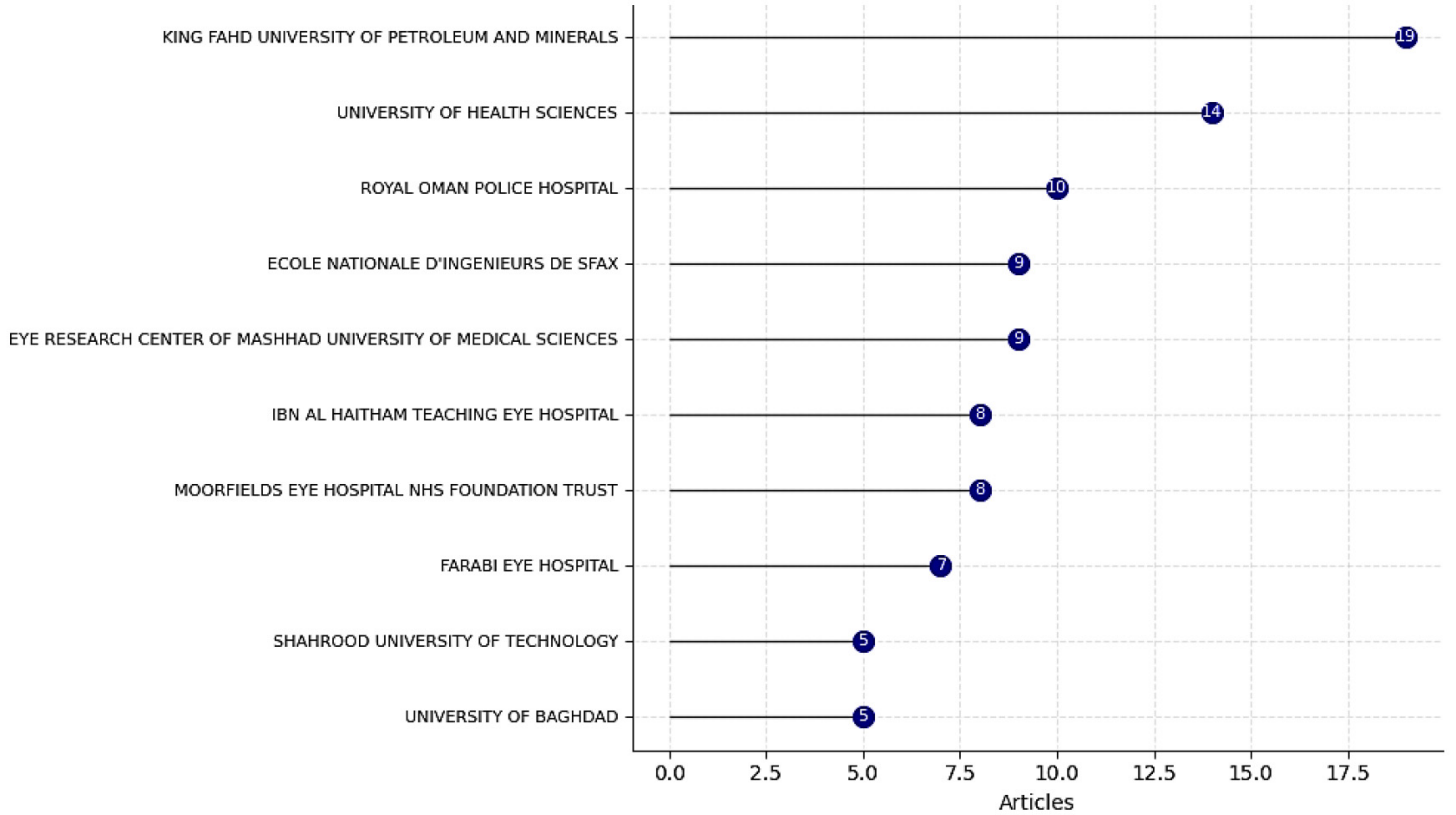
Annual Scientific Production by country related to AI in ROP Screening (2015–2025).

The geographical distribution of research output, presented in Figure 4, highlights the global engagement in leveraging technological innovation to address ROP. Türkiye, Saudi Arabia, Iran, and Egypt emerge as the most prolific contributors, within the Middle Eastern region, that collectively account for a notable proportion of publications. Their strong research productivity reflects a focused regional effort to handle critical neonatal health concerns about advances in AI based ROP.

Globally, research activity in this field extends well outside the Middle East. Notable contributions are observed from major scientific hubs in North America, particularly the United States and Canada, as well as from leading European countries such as the United Kingdom, Italy, and Germany. These outputs indicate a broad international answer of AI's potential to renovate ROP screening practices. Solid contributions from nations like South Korea further emphasizes the global nature of this scientific endeavor.

### 3.3 Countries' publications and collaboration

**Figure 5:**
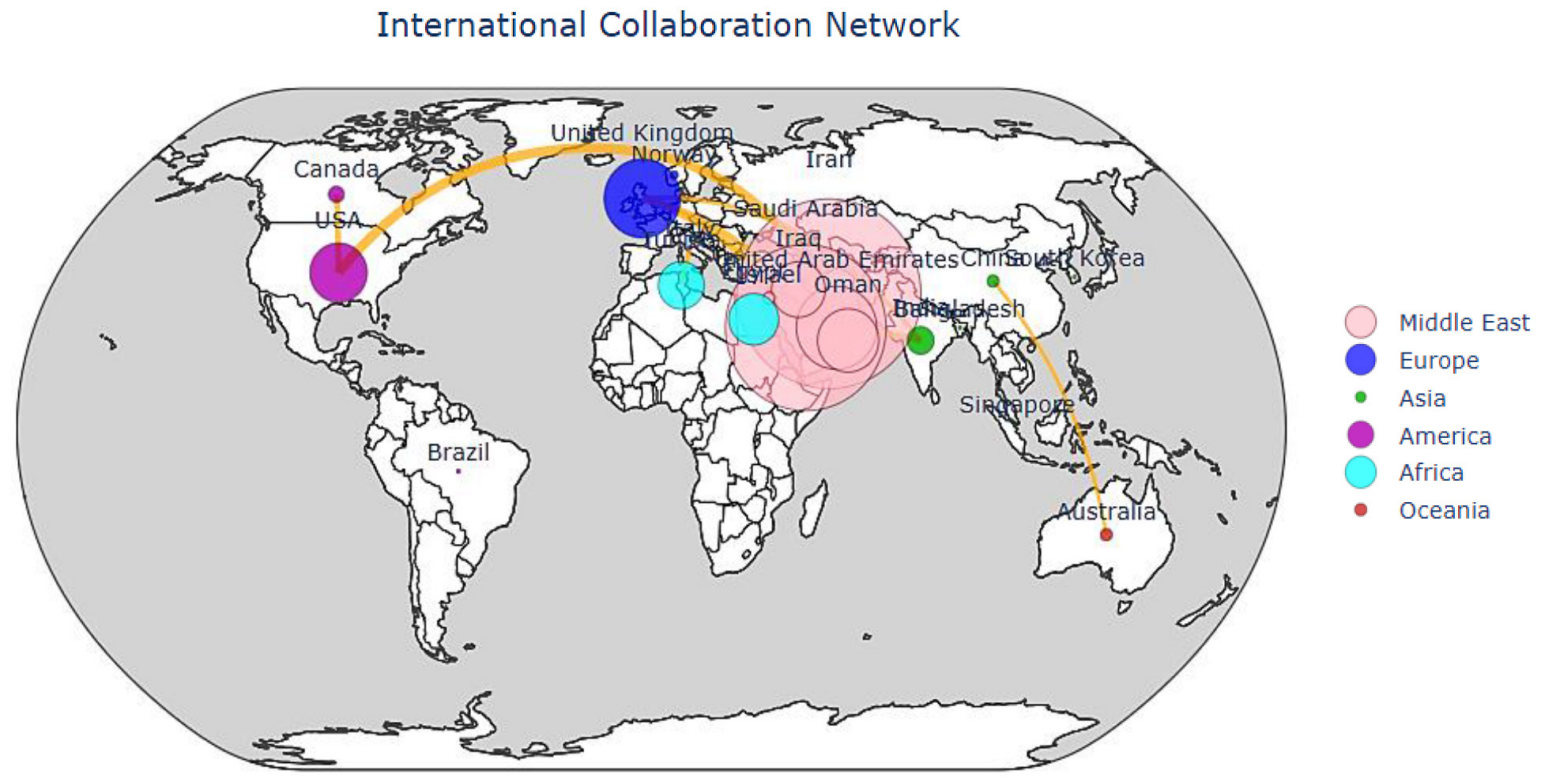
International collaboration network in AI-based ROP screening research.

The international co-authorship network illustrated in Figure 5 highlights a clear connection between high research productivity and large global collaboration. Middle Eastern countries, especially Türkiye and Saudi Arabia, are not detached entities but are central points in a sophisticated global network. The strongest collaborative links are observed between these regional centers and leading research institutions from the United States and the United Kingdom. This “South–North” collaboration reflects complementary contributions: the Middle East performs valuable clinical insights, patient datasets, and regional expertise, while Western collaborators provide innovative technical skills in AI development. These collaborations, as observed in the network structure, are associated with increased co-authorship links and broader international participation.

The visual prominence of certain countries within the collaboration network reflects higher publication output and denser co-authorship connections. While bibliometric network visualization does not directly measure causal influence or research impact quality, the observed clustering structure indicates organized and sustained international partnerships rather than isolated publication activity.

Although high-income countries such as the United States and the United Kingdom demonstrated the highest research productivity, this distribution does not necessarily reflect the global burden of retinopathy of prematurity (ROP).

ROP incidence remains disproportionately high in low- and middle-income countries (LMICs), where neonatal survival has improved but screening infrastructure and trained specialists remain limited. This mismatch between research production and disease burden highlights a translational gap.

The increasing contribution of countries such as India and China may reflect both growing neonatal populations and strategic investment in artificial intelligence solutions aimed at addressing workforce shortages. These findings suggest that AI-based screening technologies have particular relevance in resource-constrained settings, where scalable and automated diagnostic support systems could improve early detection and prevent avoidable blindness.

### 3.4 Publications per journals, publishers and institutions

The research output is communicated through a mix of high-impact, international ophthalmology journals and specialized medical AI publications. Leading publication sources include titles such BMC *Ophthalmology, Diagnostics, and Eye (Basingstoke)*, which are recognized for their rigorous peer-review processes and emphasis on translational clinical research. The distribution of studies from Q1 and Q2 journals indicates a strong positioning of this research domain within high-quality scientific outlets.

**Figure 6:**
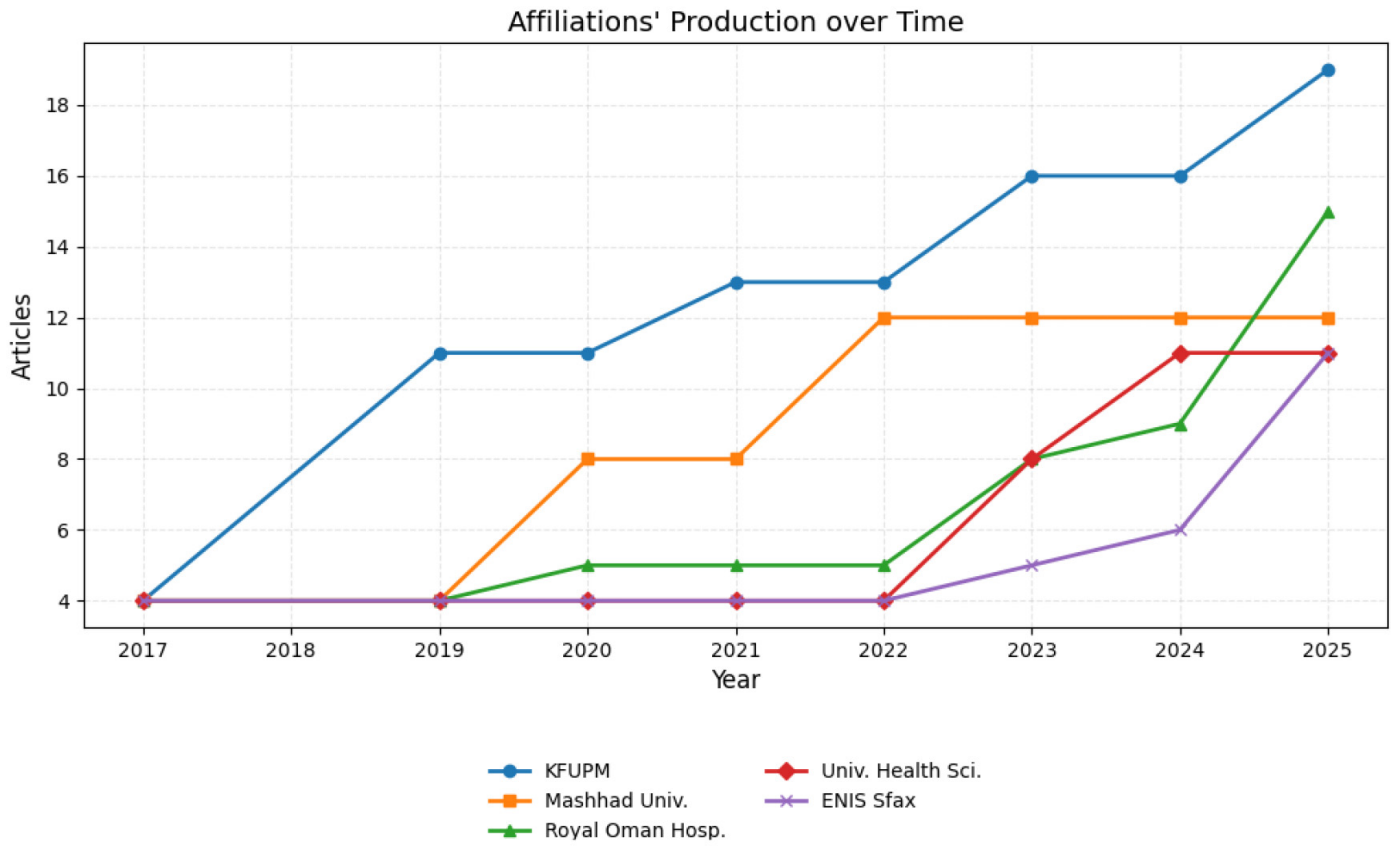
Affiliation productions over time related to AI in ROP Screening.

At an institutional level, the most productive organizations are typically large university-affiliated medical centers as shown in Figure 6, including King Fahd University of Petroleum and Minerals and the University of Health Sciences. These institutions possess the necessary three assets for success in this field: a robust department of ophthalmology with a neonatal focus, a dedicated neonatal intensive care unit (NICU) providing access to data, and a strong computer science or/and biomedical engineering department driving technical development.

### 3.5 Publications citations

**Figure 7:**
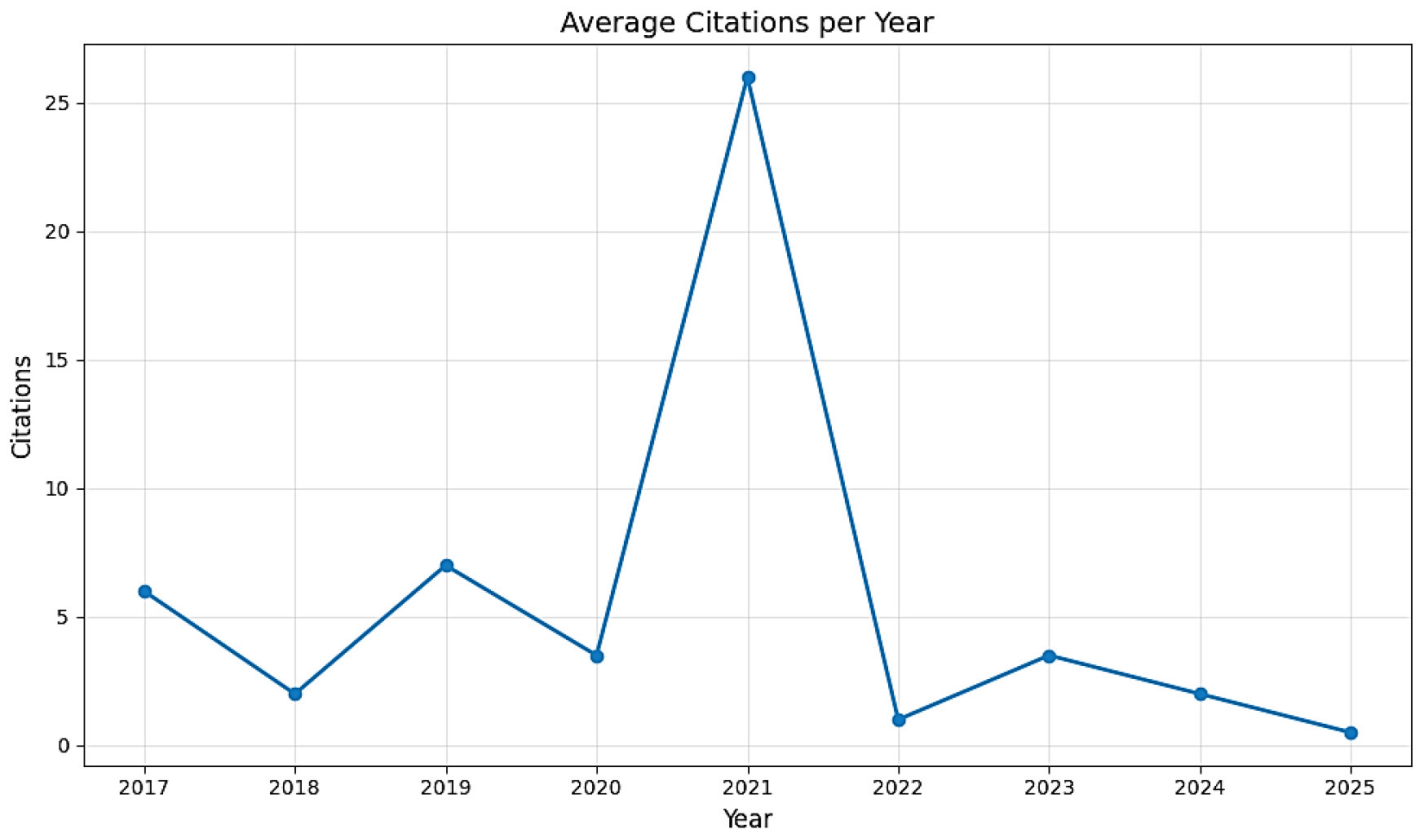
Average citation per year related to AI in ROP Screening.

Citation analysis, conducted using average citations per document and temporal citation trends, indicates measurable citation accumulation within the dataset. The dataset shows therefore an average of 18.27 citations per publication. Given the typically colored distribution of citations in bibliometric datasets, this value reflects overall citation accumulation rather than uniform impact across all documents. indicates measurable citation accumulation within the dataset; A historical examination of citation trends, shown in Figure 7, reveals a peak in average citations for papers published around 2021. This is a classic bibliometric pattern where publications have had sufficient time (3-4 years) to be read, assimilated, and cited by other researchers, yet are not old enough to be considered outdated. The subsequent lower citation counts for publications from 2023 and 2024 are simply due to their limited time in circulation and do not reduce their quality or future impact.

### 3.6 Co-occurrence of keywords

**Figure 8:**
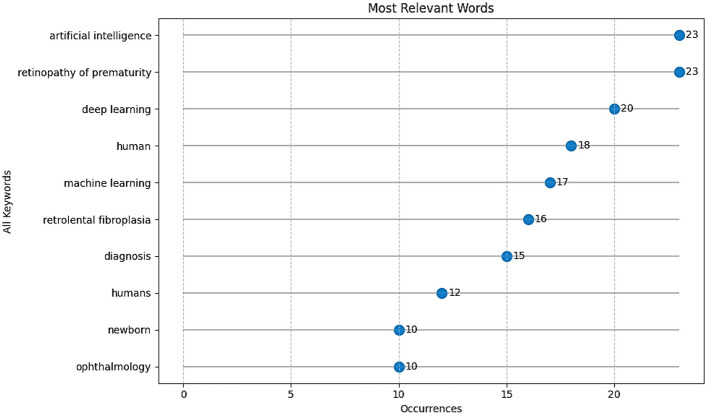
Keyword co-occurrence network related to AI-based ROP screening.

The conceptual model of this research domain, as revealed by keyword co-occurrence analysis (Figure 8), is both well-defined and interdisciplinary.

The network map highlights three prominent thematic clusters:

1) **Disease Core:** The first cluster is concentrated on the primary terms “retinopathy of prematurity,” “ROP,” and “retrolental fibroplasia,” often associated with “diagnosis,” “screening,” and “newborn.”
2) **Technology Core:** This cluster is based on methodological terms such as “artificial intelligence,” “deep learning,” and “machine learning.” The prominence of “deep learning” underscores a strong emphasis on advanced neural network approaches.
3) **Clinical Application:** The third cluster comprises terms like “ophthalmology,” “infant,” and “premature birth,” anchoring the technological research within its clinical context.

The most important connections link the disease core (“ROP”) directly to the technology core (“deep learning”, “artificial intelligence”). The keyword co-occurrence analysis illustrates a field that strategically applies sophisticated AI techniques to a clearly defined clinical challenge. The relatively solid connectivity between clusters suggests a coherent thematic structure within the research domain.

## 4 Limitations

While this bibliometric analysis offers a comprehensive overview of research on AI for ROP screening, particularly in the Middle Eastern context, several limitations are revealed. First, the study relied entirely on the Scopus database. Although Scopus is a leading and extensive multidisciplinary resource, this choice may have excluded relevant studies indexed in other databases, such as Web of Science, PubMed, or regional repositories, thus, the results may not fully capture the complete body of literature. Second, the search was limited to English-language publications. Whereas English serves as the primary language of scientific communication, this restriction may have excluded important studies published in other languages, such as Arabic, Turkish, or Farsi, that possibly underrepresenting regionally research and affecting the geographical analysis. Third, bibliometric methods usually emphasize quantifiable indicators, such as publication counts, citation metrics, and keyword occurrences which are effective for mapping the structural and temporal dimensions of the field. Meanwhile, this approach does not evaluate the scientific rigor, clinical validity, or qualitative impact of individual studies. For example, citation counts reflect influence rather than quality.

Finally, although a structured and carefully defined search strategy was applied using specific keywords and Boolean operators (e.g., “artificial intelligence,” “retinopathy of prematurity,” “screening”), the reliance on a single database (Scopus) may have limited the retrieval of studies indexed exclusively in other databases.

While Scopus provides extensive international coverage and robust citation metadata suitable for bibliometric analysis, relevant publications indexed in other sources may not have been captured. In addition, the use of predefined terminology may have excluded studies employing alternative or emerging descriptors.

Future bibliometric investigations could enhance coverage by adopting multi-database retrieval strategies, including non-English publications, and integrating quantitative mapping with qualitative assessment of highly influential studies to provide a more comprehensive representation of the field.

## 5 Implications for healthcare analytics and policy

The results of this bibliometric analysis offer several implications for healthcare analytics and policymaking in the context of AI–assisted screening for ROP. The rapid growth of AI-based ROP research identified after 2017 coincides with increasing interest in analytics-based decision-support systems in neonatal care. This study is therefore a data-driven foundation to support evidence-based prioritizing of research and development efforts in ophthalmic healthcare based on leading research themes, influential contributors, and collaboration patterns. Indeed, the dominance of deep learning and retinal image analysis highlights a clear methodological convergence that can inform technology adoption strategies within healthcare systems.

Policymakers and hospital executives can use these results to guide investments and to avoid duplication of efforts. Therefore, the identification of emerging themes related to screening accessibility and deployment in low-resource settings underscores their potential role of analytics in addressing healthcare inequalities, particularly in regions with limited access to specialized ophthalmic services.

Likewise, the detected international collaboration networks highlight the need for cross-border partnerships to accelerate innovation and transfer learning. Strategic collaboration acquired by bibliometric evidence enables capacity building and support the integration of AI-based screening tools into national ROP prevention programs especially for middle-income, developing countries, and regions with growing neonatal populations. Overall, this study highlights the potential of bibliometric analytics to inform strategic research planning to align research agendas, clinical implementation, and policy development in the evolving landscape of AI-based ophthalmic care.

## 6 Conclusions

This bibliometric mapping study provides a structured overview of the evolving research landscape at the intersection of artificial intelligence and retinopathy of prematurity (ROP) screening, with particular attention to contributions from the Middle East. The findings demonstrate sustained growth in scientific output over the past decade, highlighted by a marked increase in publications after 2018 and a peak in 2025.

The intellectual structure of the field is clearly centered on deep learning methodologies applied to retinal image analysis, reflecting a focused effort to address diagnostic challenges in neonatal ophthalmology. Collaboration patterns indicate increasing international integration, with substantial cross-regional partnerships linking Middle Eastern countries to research hubs in North America and Europe.

While bibliometric analysis does not evaluate clinical effectiveness, regulatory readiness, or implementation feasibility, the observed trends suggest growing research momentum and expanding global engagement in AI-based ROP screening. These insights may inform future research prioritization, capacity-building strategies, and strategic planning efforts, particularly in regions facing specialist shortages and screening infrastructure limitations.

Overall, AI-based ROP screening represents a rapidly developing and collaborative research domain. Continued integration of technical innovation with rigorous clinical validation will be essential to ensure that emerging technologies translate into meaningful and equitable improvements in neonatal eye care.

## Data Availability

The original contributions presented in the study are included in the article/supplementary material, further inquiries can be directed to the **corresponding author**.
